# Does Anybody Really Know What Time It Is?

**DOI:** 10.1093/function/zqaf011

**Published:** 2025-03-04

**Authors:** David M Pollock

**Affiliations:** Cardio-Renal Physiology and Medicine Section, Division of Nephrology, Department of Medicine, University of Alabama at Birmingham, Birmingham, AL 35233, USA

**Figure 1.**
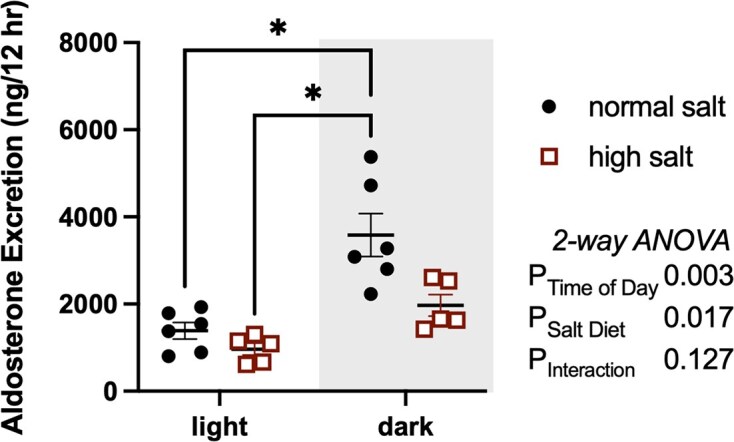
Urinary aldosterone excretion in male Sprague-Dawley rats maintained for 5-6 days on a normal (0.4% NaCl) or high (4% NaCl) diet taken in 12-h increments aligning with the light-dark cycle. Analysis was by 2-way ANOVA with Tukey’s post-hoc test to compare individual means. Adapted from Rhoads et al.^1^

How many investigators consider what time of day is most relevant for your experiments? It is clear that time of day has been overlooked as a key factor in an overwhelming number of scientific investigations and has likely slowed progress in a wide range of efforts due to factors such as conflicting findings from different laboratories and translating animal to human studies. One example of time-of-day effects, I like to use, that could mislead is the effect of salt diets on urinary aldosterone taken from a study by Rhoads et al.^1^ When comparing rats on a typical rodent chow diet versus a similar diet containing 10 times higher NaCl concentrations, the suppression of aldosterone measured in the daytime is very small to the point of not being significantly different (Figure 1). However, during the rat’s active phase at night, aldosterone is much higher in rats on a normal diet, but a high-salt diet suppresses the normal day-night difference in aldosterone.

In my own field, we have known for decades that kidney function maintains a 24-h rhythm that is independent of sleep, light, or eating times yet this has largely been ignored in the majority of biomedical research. For humans, this means that renal water and electrolyte excretion and even glomerular filtration rate (GFR) is much higher during the day compared to night. For most rodent species, on the other hand, GFR and excretory function are much lower during the day compared to night. These functional rhythms are also aligned with gene expression, enzyme activity, hormone release, and many other mechanisms that change over the course of the day and play an anticipatory role to allow for behavioral and environmental changes. The diurnal patterns in renal function align with behavioral patterns such as eating and sleeping.

Failure to recognize time-of-day-dependent changes in gene expression has likely led to incorrect or misleading conclusions. As many as 40% of genes in the liver and nearly as many in the kidney are expressed in a circadian pattern.^2^^,^^3^ Furthermore, many of the housekeeping genes and proteins used for normalization often have strong circadian rhythms themselves, such as β-actin in the kidney. While it is easy to see how new technologies such as multi-omics provide incredible advances in our understanding of the molecular basis of physiology and disease, such methods are severely limited when only one time of day is used. Furthermore, these measurements in nocturnal animals are nearly always misaligned with measurements from human studies. At a minimum, authors need to always accurately report the time of day when measurements are taken.^4^^,^^5^

The awarding of the 2017 Nobel Prize in Physiology or Medicine to Jeffrey C. Hall, Michael Rosbash, and Michael W. Young for discovering the molecular mechanisms that control circadian rhythms brought a greater awareness to this fundamental characteristic of most biological systems (https://www.nobelprize.org/prizes/medicine/2017/press-release/). The molecular clock is an autonomous transcription/translation feedback loop that runs in virtually all cells. It consists of a positive arm made up of the aryl hydrocarbon receptor nuclear translocator-like protein (Arntl, commonly referred to as Bmal1) that heterodimerizes with its partner, CLOCK (circadian locomotor output cycles kaput), to bind to E-box elements in a wide range of promoters to carry out various physiological processes. These include the expression of additional circadian genes such as Period (Per) or Cryptochrome (Cry) that eventually circle back to form the negative arm of the molecular clock by inhibiting Bmal1 and CLOCK.

Overall synchrony of these molecular clocks is largely driven by the master circadian pacemaker in the brain hypothalamus, called the suprachiasmatic nucleus (SCN), which uses its molecular clock to send output signals (through both neural circuits and paracrine signaling) to other oscillators in the brain and body.^6^ The central clock is itself regulated by light via the optic nerve, which keeps its activity aligned with the light-dark cycle. Because of the autonomous nature of the molecular clock, SCN function oscillates in constant environmental conditions, that is, the absence of light, but can be reset or shifted by environmental factors such as light, exercise, and food intake. Peripheral oscillators in other areas of the brain and body (eg, kidney, liver, vasculature, etc.) are also capable of maintaining endogenous rhythmicity but require input from the SCN for a sustained and robust synchronized rhythm over many cycles.^7^ These rhythms need to stay in synchrony for each organ system and cell within that system to anticipate its assigned function that varies according to the time of day. These secondary oscillators are also sensitive to environmental cues referred to as zeitgebers, or “time givers.” Some zeitgebers can override SCN signals such as food timing in the liver.^8^

**Figure 2.**
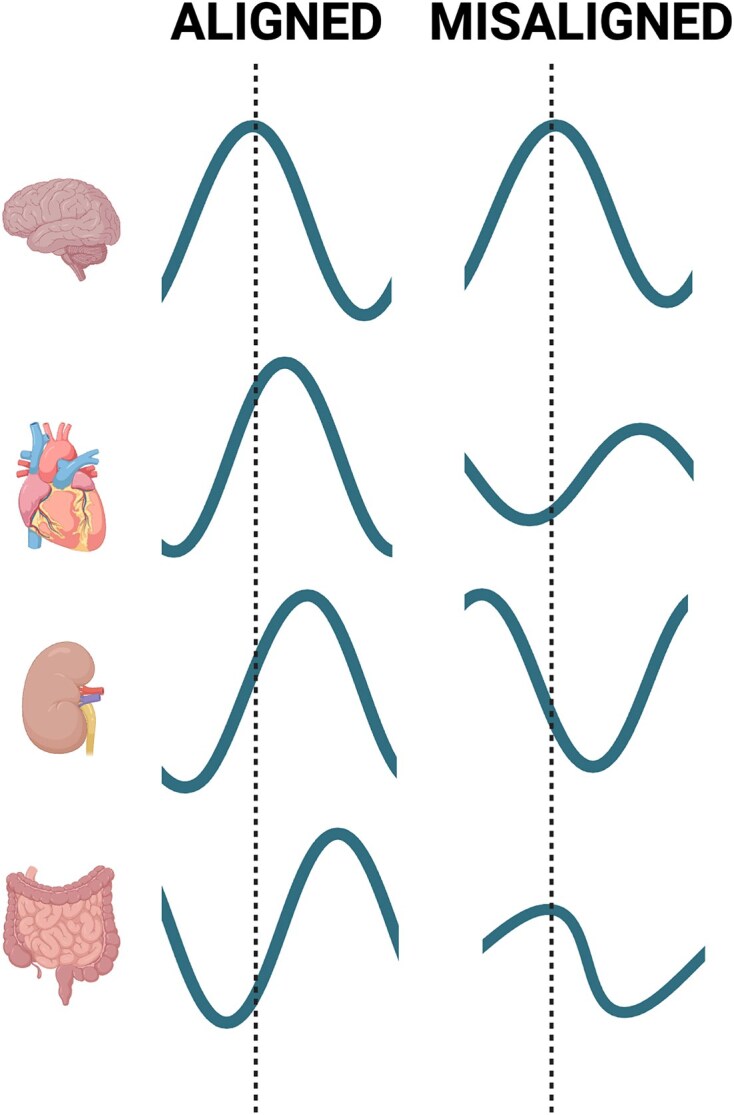
Conceptual diagram of a 24-h day-night cycle in an aligned state when rhythms are optimized for organ-specific function and a misaligned state also referred to as circadian dyssynchronization. Created in BioRender.

Circadian misalignment, or dyssynchrony, is a term used to describe situations when the rhythms of these tissue clocks are in different phases from their optimal alignment (Figure 2). For example, at night, the clocks in the gut and liver are guiding cells to rest and repair, and therefore exposure to light cues and eating places stress on areas of the body that expect sleep and fasting. Common examples of the types of circadian misalignment that are particularly important include internal physiology of organs that are out of sync with behavior or the environment, tissue-specific clocks that are out of sync between different tissues, and cell-specific clocks that are out of sync with each other within a tissue.

Because a very large number of genes, proteins, and functional activities vary by time of day, we ask the question whether much of the concern about the ability of animal models to translate to human disease could be accounted for by missing or misleading data due to only studying rodents during their subjective sleep period, while most human data are collected during their active time. One of the most useful tools now available was developed by the Hogenesch Lab, which created a database of transcriptional profiles across the circadian period.^3^ This database has proven to be a valuable resource for many in the field, but more data are needed that will be aided by the advent of newer technologies such as single-cell RNA sequencing. More recently, the team led by Dr Karen Esser has established a circadian gene expression database that expands 24-h gene expression data in a wide range of rodent tissues across the lifespan.^2^

One of the most challenging issues is trying to understand how circadian mechanisms impact physiological systems, and importantly, the pathogenesis of various diseases is the primary use of nocturnal animals. The overwhelming majority of those studies are conducted on one species, in particular the mouse. Not only are these nocturnal species, but the extremely high metabolic rate of mice also potentially changes the time frame for which functional variables may acclimate to environmental changes such as lighting, time of feeding, etc. Importantly, the molecular clock plays an essential role in regulating cellular metabolism. A good example of the problem with species selection is global knockout (KO) models for the Bmal1 gene in mice versus rats. As originally reported by the Fitzgerald Lab,^9^ Bmal1 KO mice lose their blood pressure rhythm, yet the rat model does not.^10^ Not only does this tell us something about the role of the molecular clock, it also strongly emphasizes the need for species diversity in experimental inquiry. This, however, may need to be a topic for another editorial.

**Figure 3.**
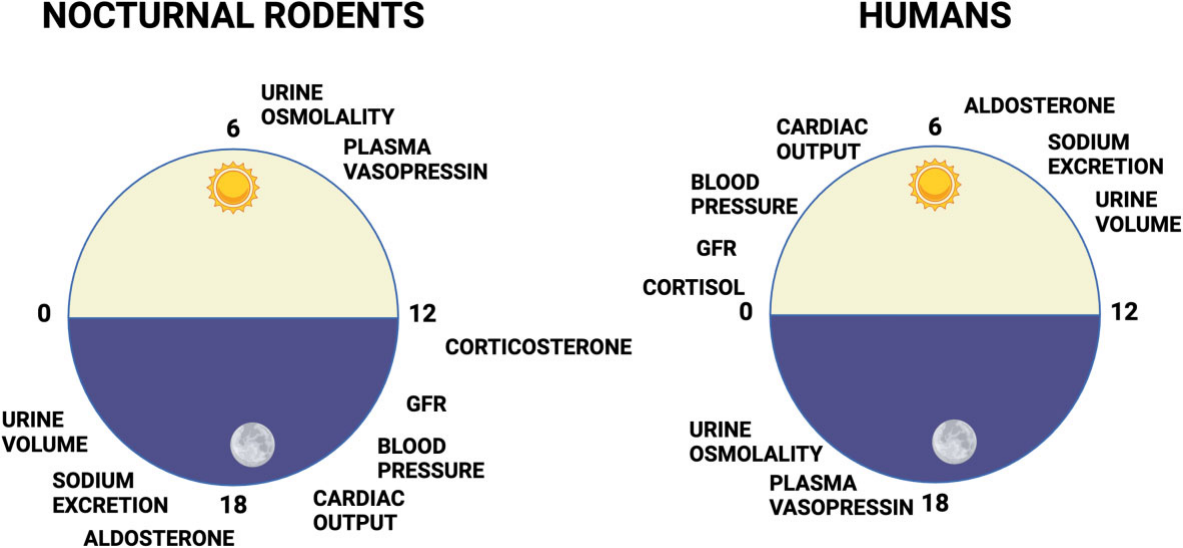
Schematic diagram of a 24-h day-night cycle showing how peak times for functional rhythms relevant to cardiovascular and renal function are at different times of day in humans compared to nocturnal animals. Created in BioRender.

For decades, we have been measuring biological functions such as blood pressure, hormone levels, neuronal activity, and countless more key end points in nocturnal animals during the day when these measures are not necessarily aligned with the time of day when they are most relevant. Figure 3 shows peak times of day for a range of cardiovascular and renal functional outputs that have strong circadian rhythms. As noted, these measures do not align in terms of time of day between nocturnal and diurnal species, yet we often do not take measurements at the optimal time of day to provide the most useful information. For example, if you are measuring blood pressure during the daytime in a mouse, the equivalent time of day in humans is the middle of the night. Given the importance of blood pressure rhythmicity in human health, measurements in rodents at a single time of day may be misleading.

Another aspect of the molecular clock that needs to be considered is the large number of sex differences observed in the relatively limited number of studies being conducted in the circadian field comparing males and females. Perhaps to no surprise, the initial animal studies have yielded contrasting effects of clock gene manipulation between the sexes.^10^^,^^11^ Once again, this is another gap in our knowledge—or rather—our approach that confounds our ability to compare and translate findings between investigations at various levels.

Herein, I was only able to highlight just a few ways in which we overlook the variable of time when we investigate biological systems. Unfortunately, circadian biology has not made its way into many areas of biomedical research, especially when it comes to mechanisms that regulate physiological variables. In most subdisciplines, such as vascular and kidney physiology, the work has been limited to a handful of labs despite these systems have extremely robust time-of-day-dependent mechanisms. Failure to consider time of day as a variable in studies in living systems has likely misled us in our quest to understand physiological systems especially when translating between species. At the very least, we need to know what time it is and the relevance of time when we study biological systems.
